# Brazilian Protocol for Sexually Transmitted Infections, 2020: infections that cause cervicitis

**DOI:** 10.1590/0037-8682-587-2020

**Published:** 2021-05-17

**Authors:** Angélica Espinosa Miranda, Mariângela Freitas da Silveira, Valdir Monteiro Pinto, Geralda Carolina Alves, Newton Sergio de Carvalho

**Affiliations:** 1 Ministério da Saúde, Secretaria de Vigilância em Saúde, Brasília, DF, Brasil.; 2 Universidade Federal de Pelotas, Programa de Pós-Graduação em Epidemiologia, Pelotas, RS, Brasil.; 3 Secretaria Estadual de Saúde de São Paulo, Programa Estadual de DST/Aids, São Paulo, SP, Brasil.; 4 Universidade Federal do Paraná, Departamento de Tocoginecologia, Curitiba, PR, Brasil.

**Keywords:** Uterine cervicitis, Chlamydia infections, Gonorrhea, Ectropion, Therapeutics, Clinical protocols

## Abstract

Infections that cause cervicitis are a topic presented in the "Clinical Protocol and Therapeutic Guidelines for Comprehensive Care for People with Sexually Transmitted Infections", published by the Brazilian Ministry of Health in 2020. The document was developed based on scientific evidence and validated in discussions with experts. This article presents epidemiological and clinical aspects of infections that cause cervicitis and recommendations on screening, diagnosis, and treatment of affected people and their sexual partnerships. Also, it discusses strategies for surveillance, prevention, and control of these infections for health professionals and health service managers involved in the programmatic and operational management of sexually transmitted infections. Expanding access to diagnostic tests and early treatment are crucial for controlling the spread of pathogens that cause cervicitis.

## Highlighted excerpt

Associated factors to cervicitis: sexually active women younger than 25 years old, new or multiple sexual partners, partners with STI, previous history or presence of other STI, and irregular use of condoms.

## INTRODUCTION

This article addresses the infections causing cervicitis, a topic that composes the Clinical Protocol and Therapeutic Guidelines (PDCT) for Comprehensive Health Care for People with Sexually Transmitted Infections (STI), published by the Health Surveillance Secretariat of the Brazilian Ministry of Health. The PDCT was developed by selecting and analyzing the available evidence from published documents and discussion in an experts’ panel. The document was approved by the National Committee for Technology Incorporation to the Brazilian National Health System (Conitec)^1^ and updated by the specialists in STI in 2020. 

## EPIDEMIOLOGICAL ASPECTS

Cervicitis, also called endocervicitis, is an STI that causes inflammation and irritation of the cervix, recognized for the first time as a critical clinical issue in 1984^2^. Their most common agents are *Chlamydia trachomatis* and *Neisseria gonorrhoeae*. However, *Trichomonas vaginalis*, *Mycoplasma genitalium*, *Ureaplasma urealiticum,* and herpes simplex virus can also cause cervicitis^3^ ^-^ ^5^. It is essential to highlight that the cervix's outer segment's inflammation, mostly related to *T. vaginalis*, gives the uterine cervix a raspberry-like aspect that does not characterize cervicitis. It is considered an extension of vaginitis called colpitis macularis, even though it is located in the cervix^6^. Mechanical or chemical irritation also causes cervicitis in which no infection is identified. The mechanical irritation sources include trauma by surgical instruments or foreign objects (pessary, diaphragm, tampon, cervical cap, or condom). The chemical irritation can be caused by exposure to latex, vaginal douche, spermicide, or contraceptive creams^7^.

In 2016, estimates of the incidence of gonorrhea in Latin-America Countries indicated that the incidence in pregnant women was 0.5% in Argentina, 2.0% in the Bahamas, 1.0% in Brazil, up to 2.0% in Colombia, and Haiti, from 2.7% to 3.0%. In non-pregnant women, in Brazil was 1.5%, in Chile, 0.6%, in Colombia, up to 0.2%; and in Haiti, from 1.0% to 4.0%. The estimate of the incidence of chlamydia in pregnant women in the Bahamas was 12.0%; in Brazil, from 9.8% to 16.7%; in Haiti, from 8.0 to 14.0%; in Chile, 5.9%, in Mexico, from 8.3% to 10.8%; and in Peru, 10.0%. In non-pregnant women, the estimate of Chlamydia incidence in Brazil was from 5.5% to 13.0%; in Chile, 8.8%; in Colombia, up to 3.2%; in Haiti, from 1.9% to 11.6%; in Mexico, 1.5%; and in Suriname, 9.5%^8^. In Brazil, there is no consolidated data at national level on the infections caused by *C. trachomatis* or *N. gonorrhoeae*, as they are not diseases with compulsory notification. A study carried out in six Brazilian states found prevalence rates of 2.1% of Chlamydia, 0.9% of gonorrhea, and 2.7% of chlamydia and gonorrhea coinfection in women living with the human immunodeficiency virus HIV^9^.In pregnant women and women looking for care in gynecology clinics^10^ ^-^ ^17^, the prevalence rates are in accordance with the ones reported by the World Health Organization (WHO).

Seventy to 80% of cervicitis cases are asymptomatic. The most typical claims are vaginal discharge, intermenstrual or postcoital bleeding, dyspareunia, dysuria, frequent urination, and chronic pelvic pain^18^ ^-^ ^23^. The risk factors are sexually active women younger than 25 years old, new or multiple sexual partners, partners with STI, previous history or presence of other STI, and irregular use of condoms^10^ ^,^ ^24^ ^-^ ^27^.

## CLINICAL ASPECTS

The symptoms of cervicitis can be similar to vaginitis, with vaginal discharge, pruritus, or dyspareunia. *C. trachomatis* and *N. gonorrhoeae* infections in women often do not produce vaginal discharge; however, if in the speculum examination, the presence of cervical mucous and cervical friability are observed, or if the swab test is positive, treatment for gonorrhea and Chlamydia must be performed, as those are the most frequent etiological agents of mucopurulent cervicitis^3^. Syndromic diagnosis of cervicitis is not effective for wide application, considering that it is asymptomatic in most cases^28^ ^-^ ^30^. The main consequences of cervicitis by Chlamydia and gonorrhea, when they are not treated, include pelvic inflammatory disease and its complications (chronic pelvic pain, ectopic pregnancy, and infertility)^18^. Cervical lesions caused by the herpes simplex virus and by syphilis can also cause cervicitis^4^. It is important to highlight that herpetic cervicitis is frequent in the primo-infection and it can be related to genital discharge^3^.

## DIAGNOSTIC

Laboratory diagnosis of cervicitis caused by *C. trachomatis* and *N. gonorrhoeae* can be done by detecting the genetic component of infectious agents by molecular biology; it is the gold standard for symptomatic and asymptomatic cases^31^. The serology for *Chlamydia* can be applied in the diagnostic investigation of previous systemic infection, such as pneumonia in newborns, lymphogranuloma venereum, salpingitis, epididymitis, infertility, and ectopic pregnancy. Still, it is not used for diagnostic of urogenital investigation^32^. The screening, in Brazil, is recommended for pregnant women younger than 30 years old in the first prenatal care appointment, people with STI, and people living with HIV at the moment of the diagnostic, in addition to victims of sexual violence and users of pre-exposure prophylaxis (PrEP) and post-exposure prophylaxis (PEP) to HIV. The inclusion of molecular biology tests for detecting *C. trachomatis* and *N. gonorrhoeae* is provided in the Brazilian National Health System (SUS) as a medium complexity procedure 02.02.03.099-7^33^.

*C. trachomatis* and *N. gonorrhoeae* infections can be diagnosed in women using first-void urine or by swab collection from the endocervix or vagina, including self-collected vaginal swabs. The nucleic acid amplification testing, NAAT are the most sensitive tests for this type of material. They are recommended for detection of *C. trachomatis* and *N. gonorrhoeae* ^30^. As an example of NAAT, we can cite the polymerase chain reaction and the transcription-mediated amplification. Examinations in other anatomic sites may be conducted in people with a history of receptive anal sex and oral sex. In *N. gonorrhoeae*, there is a possibility for culture conduction (modified Thayer-Martin selective medium). Still, this technique requires species collected with an endocervical swab, which is also available for detecting *N. gonorrhoeae* in the rectum, oropharynx, and eyes. The sensitivity of NAAT for detecting *N. gonorrhoeae* in urogenital and non-genital sites, in general, is higher than the one of culture^34^. Culture with a later test for determining the gonococcal susceptibility to antimicrobial drugs is also recommended in case of therapeutic failure^31^ ^,^ ^35^.

Laboratory diagnostic for *M. genitalium* must be ideally conducted through molecular biology tests since culture presents lower sensitivity and less convenience due to the too-long growth period^5^.

## TREATMENT

**FIGURE 1:**
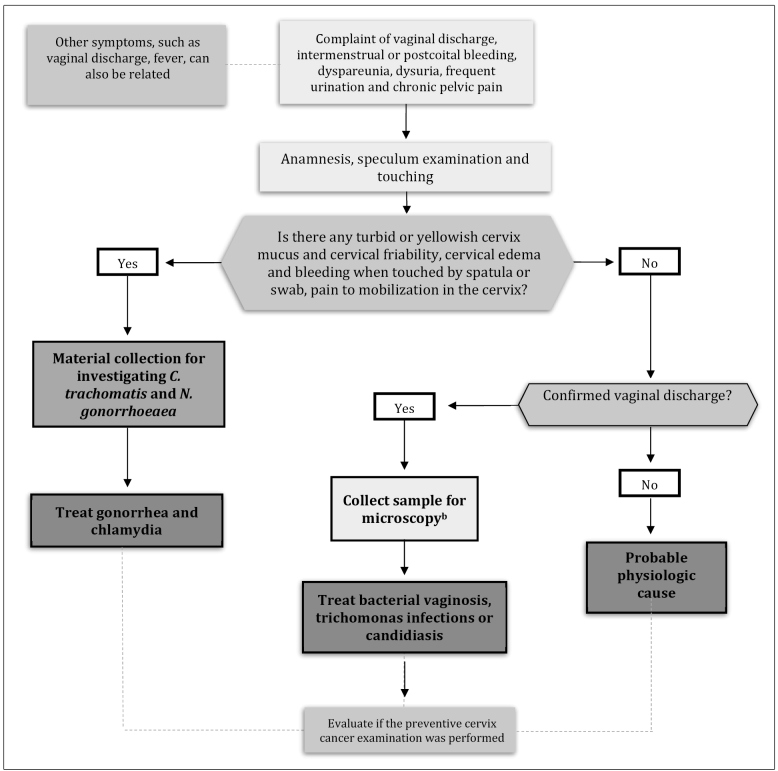
Recommendations for the management of symptomatic cervicitis

**Source:** adapted from Clinical Protocol and Treatment Guidelines for Comprehensive Health Care for People with Sexually Transmitted Infections, 2020^33^.

**Notes:** a) There are molecular biology kits detecting more pathogens simultaneously and Chlamydia and gonococcus and are also useful for diagnosing cervicitis, such as *M. genitalium*; b) Even in cases presenting normal cervical mucus and cervix, *C. trachomatis* and *M. genitalium* may be present; thus, molecular biology evidence, in case they are available, must be used to discard this possibility.

**FIGURE 2:**
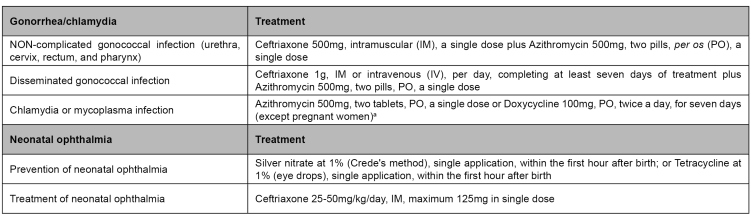
Gonorrhea and chlamydia treatment.

**Source:** adapted from Clinical Protocol and Treatment Guidelines for Comprehensive Health Care for People with Sexually Transmitted Infections,2020^33^.

**Notes:** a) In cases of mycoplasma, azithromycin is preferred, as doxycycline presents high levels of resistance; b) Local instillation of physiological solution, each hour; local penicillin instillation is recommended; in unsatisfactory therapeutic response cases, consider the hypothesis of simultaneous chlamydia infection.

The clinical management of cervicitis cases is important for its effective control. The recommendations for the management of cervicitis, with a description of clinical routine, are presented in Figure 1. The drugs and regimes of treatment recommended^33^ are described in Figure 2.

Like the Brazilian protocol, the North-American one recommends, as a treatment for *C. trachomatis*, azithromycin 1g, *per oral* (PO), in a single dose, or doxycycline 100mg, PO, twice a day, for seven days (not recommended for pregnant women)^33^ ^,^ ^35^.In cases of non-complicated infections in the cervix, urethra, and rectum caused by *N. gonorrhoeae*, the drug regime recommended by the PDCT is ceftriaxone 500mg, intramuscular (IM), in a single dose, plus azithromycin 1g, PO, in a single dose, administered preferably simultaneously^33^. The recommended dose of ceftriaxone 500mg made by Conitec^34^ was based on an assessment of cost-benefit regarding the use and financial impact of ceftriaxone 250mg in the country. Different doses of ceftriaxone, such as 250mg^35^, are recommended in other countries, according to drug availability and local data on drug susceptibility.

A challenge in treating *N. gonorrhoeae* is the increase of strains presenting clinical resistance to antimicrobial drugs, including cephalosporins, tetracycline, quinolones, and penicillins. Likewise, another agent that has shown high resistance to drugs is mycoplasma. Although the first-line treatment chosen is azithromycin, studies have shown up to 30% resistance in some places^4^.

Symptoms persisting after the treatment of *N. gonorrhoeae* must be assessed through culture and test for determining the susceptibility of gonococcus to the antimicrobial drugs. We highlight that other microorganisms may also cause persistent cervicitis. Therapeutic failure must be considered for people who continue to present symptoms three to five days after treatment and without sexual contact within such period, as well as for people with a positive cure test (positive culture after 72 hours or positive NAAT at least seven days after the treatment), in case there is no report of sexual contact within such period^33^.

## SURVEILLANCE, PREVENTION, AND CONTROL

As cervicitis is usually caused by an IST, it is essential to reinforce using condoms in all sexual intercourses. If a person is diagnosed with an STI, their recent sexual partners must also be tested and treated with the scheme aforementioned^33^.

Patients with positive tests must be advised to refrain from sexual contact for seven days after the treatment and resolve possible symptoms^33^ ^,^ ^35^. It is essential to offer information on the infections, including details on transmission, prevention, and complications, and advising all sexual partners^33^. The provision of verbal and written information, in addition to testing for other STI, such as gonorrhea, syphilis, HIV, and hepatitis B, among others, is recommended.

Sexual contacts must be encouraged to take tests, in addition to receiving advice and treatment for Chlamydia infection and other STI^35^ ^-^ ^39^. All sexual contacts of the six months before starting symptoms or performing diagnostic must ideally be evaluated, tested, and treated^37^ ^,^ ^40^.

The tests for control of cure of non-complicated urogenital or rectal *C. trachomatis* and *N. gonorrhoeae* infections are not routinely recommended for people treated with first-line schemes. However, they can be conducted during pregnancy, in cases of complicated infections or persistence of symptoms^32^. In situations of Chlamydia infection, extragenital infections may also be considered for investigation, mainly when azithromycin has been administered for treatment of rectal infections^38^.

When recommended, the test for cure control for Chlamydia should be performed four weeks after completion of therapy and through molecular tests^37^ ^,^ ^38^. The control test for detection of reinfection within three to six months may be ideally offered to young women and men (younger than 25 years old), presenting positive results for *C. trachomatis* ^33^ ^,^ ^37^.

*C. trachomatis*, *M. genitalium,* and *N. gonorrhoeae* are not on the list of compulsory notification diseases of the Brazilian Ministry of Health, but the states and local governments can notify them.

Regarding *N. gonorrhoeae*, due to the development of high resistance to antimicrobial drugs, WHO has a program for worldwide surveillance of the gonococcal susceptibility to drugs, the Gonococcal Antimicrobial Surveillance Program (GASP). Brazil participates in this program, through the SenGono (Gonococcal Sensitivity) Project, which carries out this surveillance at the national level from samples of male urethral discharge, as recommended by WHO^41^. In a complementary way, within the scope of SenGono Project, the etiological agents of male urethral discharge are also being researched, which are the same pathogens present in cases of cervicitis - through the molecular tests performed with biological samples collected in all sites of the project^42^.

## SPECIAL POPULATIONS

### Pregnant women and newborns

The gonococcal and Chlamydia infections during pregnancy may be related to preterm births, premature rupture of membrane, fetal losses, intrauterine growth retardation, and postpartum endometritis, in addition to newborn conjunctivitis and pneumonia^43^ ^-^ ^46^.

In newborns, the main clinical manifestation is conjunctivitis, and there can be septicemia, arthritis, scalp abscesses, pneumonia, meningitis, endocarditis, and stomatitis^47^. Neonatal ophthalmia occurs in the first month of life, and, in case it is not treated, it can lead to blindness, mostly when caused by *N. gonorrhoeae*. For this reason, the disease must be treated immediately to prevent eye damage. Usually, the newborn is taken to the health service due to eyelids erythema and edema and conjunctive or mucopurulent material in the eyes. Chlamydia conjunctivitis is much less severe, and its incubation period ranges from five to 14 days^33^. The relative frequency of eye infection by both etiologic agents depends on their prevalence in pregnant women and on the use of eye prophylaxis within an hour after birth^33^.

When available, research must be carried out for *N. gonorrhoeae* and C. trachomatis through molecular biology in a first prenatal medical appointment. The treatment is recommended for non-complicated urogenital infection by *C. trachomatis* during pregnancy and breastfeeding. The test for control of cure must also be conducted if it is available. Azithromycin was considered safe and efficient according to clinical experience, and WHO also recommended it during pregnancy^33^.

### HIV infections

STI are considered one of the main factors facilitating HIV transmission. The HIV infection changes the natural course of many infections, increasing their duration and making them more resistant or making them more recurrent and keeping a synergy between the HIV infection and other STI^48^ ^,^ ^49^. The cervix is a common and well-documented place for HIV transmission. The invasive intracellular pathogeny of *C. trachomatis* may cause substantial damages to the endocervical epithelium, making infection by HIV infection easier^50^.

According to international studies, chlamydia infection prevalence in women living with HIV may vary from 2.0% to 10.0%^51^ ^-^ ^53^ up to 18.1%^52^. A study in Thailand showed a prevalence rate of 9.7% for Chlamydia among 824 women infected with HIV^54^. In Brazil, the prevalence can vary between 2.1% and 17.6%^8^ ^,^ ^55^ ^-^ ^57^, depending on the place, the diagnostic method and sample type used. Regarding *N. gonorrhoeae*, the molecular mechanism associated with the increase of HIV transmission induced by the gonococcus is still not widely defined due to a proper in vitro model's unavailability. However, there is a hypothesis that this bacterial infection promotes the increased recruitment of endocervical CD4% T cells, providing more targets for the activation of HIV-1^58^. The prevalence of gonorrhea in Brazil varies from 0.5% to 0.9% in this group^8^ ^,^ ^55^.

Chlamydia and gonorrhea infections may present a more severe evolution and higher indexes of complications when they occur in women living with HIV^59^ ^-^ ^61^.

A cohort study conducted in women infected with HIV who have cervicitis by *M. genitalium* showed a prevalence of 7.4%, indicating that *M. genitalium* is a frequent coinfection in women living with HIV^62^. The association between HIV infection and the infections causing cervicitis, in addition to the combined epidemiology, takes place partially due to the fact that these STI have common sexual risk behavior factors, such as multiple sexual partners^61^. The criteria for diagnosing and treating cervicitis for people living with HIV are the same ones used in those without HIV^63^.

## ADOLESCENTS

Adolescents present higher risk of getting STI, including *N. gonorrhoeae* and *C. trachomatis* infections, both from behavioral and biological perspectives. Adolescents are more likely to risky sexual behavior, such as simultaneous partners or sexual intercourses without condoms. Besides, adolescents present a lower probability of accessing and using sexual health services in comparison with adults^64^ ^,^ ^65^. Such factors lead to a higher chance of exposure and a lower likelihood of diagnostic and treatment. From the biological point of view, female adolescents are particularly susceptible to STI due to the lower production of cervical mucus and increased cervical ectopy^66^. Therefore, in case they are exposed to an STI, adolescents are more likely to get infected than adults, as the columnar epithelium does not have the immunological defense ability of epithelial cells^64^ ^,^ ^65^. The criteria for diagnosing and treating cervicitis for adolescents are the same in women in general^64^.
